# Medication-related challenges in patients living with an ostomy: Insights from a national cross-sectional exploratory survey

**DOI:** 10.1016/j.rcsop.2026.100797

**Published:** 2026-05-12

**Authors:** Stefanie Hehenberger, Sonja Glamočlija, Irene Lagoja

**Affiliations:** a Klinik Ottakring, Hospital Pharmacy, Montleartstraße 37, 1160 Vienna*,* Austria; b Vienna Doctoral School of Pharmaceutical, Nutritional and Sport Sciences, University of Vienna, Josef-Holaubek-Platz 2, 1090 Vienna, Austria; c Department. of Pharmaceutical Sciences, University of Vienna, Josef-Holaubek-Platz 2, 1090 Vienna, Austria

**Keywords:** Ostomy, Ileostomy, Colostomy, Medication safety, Drug absorption, Patient education, Pharmacotherapy

## Abstract

**Objectives:**

Patients with an ostomy may experience altered drug absorption and efficacy due to gastrointestinal changes, potentially leading to medication-related safety risks. This study aimed to explore patient-reported experiences with oral medication therapy and to identify gaps for improved counseling.

**Methods:**

A national cross-sectional exploratory online survey was conducted among adults (≥18 years) with an ileostomy or colostomy in Austria between June and October 2025. A study-specific questionnaire was developed based on literature review and expert input, addressing perceived medication effectiveness, observation of undigested dosage forms, and communication with healthcare professionals. Descriptive statistics, chi-square tests/ Fisher's exact test, and binary logistic regression analyses were performed.

**Results:**

Fifty participants provided information on ostomy type (58.0% ileostomy, 42.0% colostomy). Among respondents, 35.8% reported observing undigested tablets or capsules in their ostomy bag, with significantly higher rates in ileostomy patients (55.2%) compared with colostomy patients (14.3%; *p* = .003). Perceived changes in medication effectiveness were reported by 29.2% of participants, again with significantly higher prevalence among ileostomy patients (42.9% vs. 10.0%; *p* = .014). Only 24.5% of respondents had received professional counseling on potential medication-related changes after ostomy formation. Logistic regression analyses suggested that ostomy type (OR = 0.094, 95% CI: 0.017–0.527) and lack of professional information (OR = 0.083, 95% CI: 0.008–0.807) were associated with perceived medication-related problems.

**Conclusion:**

A substantial proportion of ostomy patients experience medication-related problems, including undigested dosage forms and perceived changes in drug effectiveness. Ileostomy patients are particularly vulnerable. The findings indicate potential gaps in patient education and communication with healthcare professionals. Systematic medication review after ostomy formation, proactive counseling on high-risk dosage forms, and improved interprofessional collaboration may represent important strategies to enhance medication safety in this vulnerable population.

## Highlights

• Individuals living with an ostomy are a vulnerable population regarding medication-related problems.
• Ileostomy patients are particularly at risk for altered drug absorption.
• Systematic medication review is needed after stoma formation.
• Structured counseling and documentation improve safe medication use.

## 1 Background

The term “ostomy” derives from the Greek word “stoma” (στόμα), meaning “mouth.” In medicine, an ostomy refers to a surgically created opening of a hollow organ on the body surface to enable excretion of waste products. An enterostomy is a surgically fashioned intestinal opening. Intestinal ostomies are classified according to the segment of intestine brought to the body surface: small-bowel ostomies (ileostomies) can be distinguished from large-bowel ostomies (colostomies).^1^

Official data on the prevalence of individuals living with an ostomy are scarce. In Europe, approximately 700,000 individuals are estimated to live with an ostomy.^2^ In Austria, specific national prevalence data are not available according to Statistics Austria (written correspondence, January 2026); however, it is estimated that approximately 15,000 people are living with an ostomy nationwide.^3^, ^4^ In addition, around 1500 colostomies and 700 ileostomies are performed annually in Austria.^5^

In adults, stomas are most commonly created for the management of colorectal cancer, inflammatory bowel disease, diverticular disease, bowel obstruction, or to protect distal anastomoses, with colorectal cancer being the leading indication.^6^, 7. Although ostomy surgery can be life-saving, it is associated with a substantially impaired health-related quality of life. As a life-altering intervention, it affects multiple domains of daily living, while persistent physical challenges, psychological distress, and ostomy-related complications further hinder adaptation and self-management, underscoring the need for adequate patient education, follow-up, and individualized management strategies.^8^, ^9^, 10., 11. Beyond these well-recognized challenges, ostomy formation also has important implications for pharmacotherapy.

Gastrointestinal structure and function are critical determinants of oral drug absorption. Surgical interventions such as ostomy formation or bowel resection can alter drug absorption, bioavailability, and therapeutic effectiveness.^12^ Medication effectiveness in ostomy patients may be influenced by multiple factors, including ostomy type, remaining bowel length, underlying disease, and the physicochemical properties of medications.13., 14. These physiological alterations are often insufficiently recognized by clinicians and may have significant implications for pharmacotherapy.^15^, ^16^ Having an ostomy typically does not involve the use of specific medications; however, underlying diseases often require pharmacological treatment, and polypharmacy (>5 medications) is common among individuals living with an ostomy.^17^

Awareness of medication-related problems in patients living with an ostomy remains limited among both patients and healthcare professionals.^13^ Both ileostomy and colostomy are associated with considerable morbidity, and outcomes may be negatively affected by inconsistent assessment and insufficient communication with healthcare providers.^18^

These communication gaps are particularly relevant in the context of medication safety, which encompasses the prevention and management of medication-related errors and harm throughout the entire medication journey, from prescribing to monitoring and follow-up.^19^, ^20^ In recent years, the involvement of patients in the prevention of medication errors has gained increasing attention.^21^ Patient engagement and patient-reported experiences are positively associated with clinical effectiveness and patient safety and are recognized as central pillars of healthcare quality.^22^ Actively involving patients in medication management may therefore represent an important strategy to identify safety risks, enhance communication, and reduce preventable harm.

Despite established knowledge about physiological changes affecting drug absorption after ostomy formation, systematic data on patient experiences, perceptions, and challenges regarding medication use remain scarce. Given the physiological alterations affecting drug absorption and the potential risk of medication-related problems, there is a compelling need for patient-centered research in this population. Such research is essential to identify the frequency and nature of medication-related problems from the patient perspective, assess information needs, and evaluate communication with healthcare professionals. These data are indispensable for developing targeted interventions to optimize pharmacotherapy, enhance medication safety, and ultimately improve health-related quality of life in patients living with an ostomy. The aim of this study was to assess patient-reported experiences with oral medication therapy, including perceived changes in medication effectiveness, observation of undigested dosage forms, and quality of information received from healthcare professionals among individuals living with an ostomy, to identify information gaps and opportunities for improving patient counseling and medication safety in clinical practice.

## 2 Method

### 2.1 Study design

A national cross-sectional exploratory online survey was conducted among individuals living with an ileostomy or colostomy in Austria.

### 2.2 Questionnaire development

As no validated questionnaire addressing the study objectives was available, a study-specific instrument was developed. Items were generated based on an extensive literature review on questionnaire methodology and patient safety in medication therapy, informed by a previously published questionnaire in a comparable context and refined through thorough discussion among the co-authors.^23^ The instrument comprised 16 items, including closed-ended, semi-open, and open-ended questions. It was designed to capture patient-reported experiences related to perceived medication effectiveness, observation of undigested tablets or capsules, use of alternative dosage forms, communication with healthcare professionals, medication tolerability and additional challenges concerning pharmacotherapy. The draft questionnaire underwent pretesting with two hospital pharmacists and one patient with an ostomy. Feedback from the pretest was used to refine item wording, improve clarity and unambiguity, and assess feasibility and technical functionality. No substantial structural changes were required after pretesting. The questionnaire was originally developed in German and administered exclusively to German-speaking participants; therefore, no translation process was required.

As this was an exploratory study and the questionnaire did not include multi-item scales measuring a single latent construct, formal psychometric validation (e.g., internal consistency testing using Cronbach's alpha) was not performed.

A detailed description of all questionnaire items is provided in Supplementary File 1.

### 2.3 Data collection

Data were collected between June and October 2025. The anonymous survey was administered using SoSci Survey, a GDPR-compliant online platform.

### 2.4 Participants and recruitment

Participants were recruited via patient self-help groups in Austria. These organizations exclusively support individuals living with an ostomy. Initial contact was established via publicly available organizational contact information.^24^ Group coordinators were approached and informed about the study objectives and procedures, and they subsequently distributed the study invitation and survey link to their members. Eligible participants were adults (≥18 years) living with an ileostomy or colostomy and with sufficient proficiency in the German language to understand and complete the questionnaire independently.

### 2.5 Data analysis

No formal sample size calculation or power analysis was performed, as this study was designed as an exploratory cross-sectional survey with a descriptive and hypothesis-generating purpose. Statistical analyses were performed using IBM SPSS Statistics (Version 31). Both descriptive and inferential statistical methods were applied.

Descriptive analyses were conducted using frequency tables to characterize the study population with respect to ostomy type, sex, and regular medication use. Descriptive statistics were calculated using variable-specific casewise exclusion, allowing the maximum number of valid responses to be included for each analysis.

Group comparisons (ileostomy vs. colostomy) were performed using chi-square tests with Phi or Cramér's V as effect size measures to identify significant differences in categorical variables. For cells with expected frequencies <5 in more than 20% of cases, Fisher's exact test was applied instead of Pearson's chi-square test. Binary logistic regression models were calculated to examine the influence of selected predictors on the dependent variables: (1) perceived medication effectiveness and (2) occurrence of undigested dosage forms in the ostomy bag. Independent variables included ostomy type, age, sex, information provided by healthcare professionals, and communication with healthcare providers. Model fit was evaluated using Omnibus Tests of Model Coefficients, classification tables, and pseudo-R^2^ indices (Cox & Snell and Nagelkerke). For the analysis examining the association between dosage form variables and perceived medication effectiveness, Firth's penalized likelihood logistic regression was applied to address potential bias due to small sample size and rare events. It should be noted that some models included fewer than the recommended 10 events per predictor variable, which may affect estimate stability.

Exploratory analyses were conducted to examine associations between information provided by healthcare professionals and clinical outcomes using cross-tabulations and chi-square tests or Fisher's exact tests, as appropriate.

The level of statistical significance was set at *p* < .05. For inferential analyses, missing values were excluded listwise.

Open-ended responses were analyzed qualitatively using thematic categorization and content analysis to identify patient safety–relevant themes.

### 2.6 Methodological quality and validity

Online administration ensured full standardization of data collection, with all participants receiving identical questions in the same order, thereby supporting procedural objectivity. Automated data capture and export minimized transcription errors and supported analytical objectivity.

Internal consistency was promoted through clear and unambiguous item formulation. Content validity was ensured through a systematic review of relevant literature, integration of clinical expertise from two hospital pharmacists, and incorporation of experiential input from a patient with an ostomy. The positive assessment of content, structure, and clarity during pretesting further supported content validity.^25^

## 3 Results

### 3.1 Study population and ostomy characteristics

Depending on the item, between 25 and 53 participants provided valid responses. Information on ostomy type was available for 50 participants (Table 1). Of these, 29 (58.0%) reported having an ileostomy and 21 (42.0%) reported having a colostomy. Sex was reported by 49 participants; the majority were female (*n* = 37, 75.5%), while 12 (24.5%) were male.

**Table 1:** Baseline characteristics of study participants.

Variable	N valid responses	Result n (%) / Median (Range)
Ostomy type (Ileostomy / Colostomy)	50	29 (58.0%) Ileostomy, 21 (42.0%) Colostomy
Sex (female / male)	49	37 (75.5%) female, 12 (24.5%) male
Regular medication use (Yes / No)	48	35 (72.9%) Yes, 13 (27.1%) No
Number of daily medications	27	6 (Range: 1–16)

### 3.2 Medication use and therapy changes

Of the 48 participants who responded to the question on regular medication use, 35 (72.9%) reported taking medications regularly, while 13 (27.1%) reported no regular medication use. 27 participants (56.3%) provided additional information on the number of daily medications. Among these, the median number of daily medications was six (range: 1–16).

Regarding changes in medication after ostomy formation, 49 participants provided valid responses. The majority (*n* = 35, 71.4%) reported no changes in their medication regimen, whereas 14 (28.6%) reported changes. Reported modifications included discontinuation or dose reduction of medications, switching of active substances, and changes in dosage forms. These data were obtained from free-text responses, with multiple entries possible per participant.

### 3.3 Perceived changes in medication effectiveness

Of the 48 participants who responded to the question on perceived medication effectiveness, 14 (29.2%) reported changes in medication effects following ostomy formation, while 34 (70.8%) reported no perceived changes. Reported changes included reduced or absent therapeutic effects, fluctuating or elevated blood pressure values, altered thyroid hormone levels, and the presence of undigested tablets or capsules in the ostomy bag. Several participants reported switching from solid oral dosage forms to liquid formulations or drops.

Representative patient statements included:

*“The medication has hardly any effect anymore…” (P11)*

*“I had to switch from tablets to drops because the tablets were excreted undigested…” (P14)*

*“Tablets and capsules are absorbed poorly since the* ostomy *surgery…” (P17)*

*“My thyroid hormone levels are extremely lower than before…” (P20)*

*“My blood pressure is higher than before [the* ostomy *formation] …” (P29).*

A statistically significant difference was observed between patients with an ileostomy and those with a colostomy regarding perceived medication effectiveness. Specifically, 42.9% (12/28) of ileostomy patients reported a change in medication effectiveness compared with 10.0% (2/20) of colostomy patients (χ^2^ test: *p* = .014, φ = −0.356).

### 3.4 Information provided by healthcare professionals

Of the 49 participants who responded, 12 (24.5%) reported receiving information from healthcare professionals regarding potential changes in medication effectiveness after ostomy formation, while 37 (75.5%) reported receiving no such guidance. Reported sources included physicians, pharmacists, and, in some cases, a combination of physicians and ostomy care nurses. No significant difference was observed between ileostomy and colostomy patients regarding receipt of professional information (Fisher's exact test: *p* = .313).

In addition, 15 of 48 participants (31.3%) reported having discussed potential medication-related changes with their general practitioner or specialist after ostomy formation, while 33 (68.7%) reported no such discussion.

Among the 48 participants with complete data, perceived changes in medication effectiveness were reported by 36.1% (13/36) of those who had not received professional information, compared with 8.3% (1/12) of those who had received such information. This difference did not reach statistical significance (Fisher's exact test *p* = .081).

### 3.5 Medication handling and observations in daily life

Of the 49 participants who responded, 18 (36.7%) reported regularly checking their ostomy bag for medication residues, while 31 (63.3%) did not. No significant difference was observed between ileostomy and colostomy patients in checking behavior (χ^2^ test: *p* = .694, φ = 0.056). Overall, 19 of 53 participants (35.8%) reported having observed undigested tablets, capsules, or other dosage forms in the ostomy bag at least once.

When stratified by ostomy type, 16 of 29 ileostomy patients (55.2%) reported finding undigested medication residues compared with 3 of 21 colostomy patients (14.3%). This difference was statistically significant (χ^2^ test: *p* = .003, φ = −0.416).

Among the 19 participants who reported undigested medication residues, 5 (26.3%) were able to identify the specific medication. In 8 cases (42.1%), the observation resulted in therapy modification, including switching to liquid formulations, drops, injections, or discontinuation of solid oral medications.

Among the 48 participants with complete data, those who detected undigested dosage forms reported perceived changes in medication effectiveness more frequently (50.0%, 7/14) than those who did not (20.6%, 7/34). However, this association was not statistically significant (Fisher's exact test *p* = .078).

### 3.6 Communication with healthcare providers and pharmacies

Among the 49 participants who responded, 39 (79.6%) reported informing physicians about their ostomy during medical consultations, while 10 (20.4%) did not. When purchasing medications at a pharmacy, 21 of 49 participants (42.9%) reported informing pharmacy staff about their ostomy, whereas 28 (57.1%) did not.

When asked whom they would consult regarding medication-related questions (*N* = 53, multiple responses allowed), 38 participants (71.7%) indicated physicians, 20 (37.7%) indicated pharmacists, 6 (11.3%) indicated nursing staff, and 5 (9.4%) indicated other sources.

### 3.7 Dosage forms and medication tolerability

Of the 49 participants who responded, 14 (28.6%) reported having tried or been recommended alternative dosage forms other than tablets. These included liquid medications, drops, suspensions, orally disintegrating tablets, sprays, patches, and injectable therapies.

Among the 48 participants with complete data, dosage form changes (e.g., switch to liquid formulations) were significantly associated with perceived changes in medication effectiveness. Specifically, 85.7% (12/14) of participants with dosage form changes reported altered effectiveness, compared with 20.6% (7/34) of those without changes (χ^2^ test: *p* < .001, φ = 0.605).

Medication intolerance or avoidance since ostomy formation was reported by 6 of 46 participants (13.0%). The most frequently mentioned substance was magnesium, particularly oral or effervescent formulations, due to diarrhea. Isolated reports involved specific antidepressants and rectal dosage forms.

### 3.8 Additional experiences and challenges

In open-ended responses (*N* = 54 entries from 25 participants, multiple responses allowed), participants reported challenges related to insufficient information, uncertainty regarding actual drug absorption, difficulties with solid oral dosage forms, management of diarrhea, and maintaining adequate vitamin and electrolyte levels. Several participants indicated that they had never been explicitly informed about potential medication-related implications associated with having an ostomy.

Representative patient statements included:

*“Sometimes medications have to be crushed [to improve absorption] …” (P3)*

*“There is uncertainty about how much active substance is actually absorbed. Even specialists say they do not really know…” (P15)*

*“Getting medications, for example as orally disintegrating tablets, can be difficult …” (P17)*

*“Nobody feels responsible [for providing guidance about my medications] …” (P41)*

*“I did not receive any information [from healthcare professionals] …” (P49).*

### 3.9 Logistic regression analyses

Perceived medication effectiveness*:* ostomy type emerged as a strong predictor (*p* = .007, OR = 0.094, 95% CI: 0.017–0.527), indicating that ileostomy patients had higher odds of reporting altered medication effectiveness compared with colostomy patients. Information provided by healthcare professionals was also a significant predictor (*p* = .032, OR = 0.083, 95% CI: 0.008–0.807); patients who had not been informed about potential medication-related changes showed increased odds of reporting medication-related problems (Table 2). Age and sex were not significantly associated with perceived medication effectiveness, indicating no observable differences between male and female participants or across different ages (age: *p* = .147; sex: *p* = .204).

**Table 2:** Logistic regression analyses identifying predictors of medication-related problems in patients living with an ostomy.

Outcome	Predictor	Odds ratio (OR)	95% CI	*p*-value
Perceived medication effectiveness	Ostomy type (ileostomy vs. colostomy)	0.094	0.017–0.527	0.007
	Information provided by healthcare professionals (no vs. yes)	0.083	0.008–0.807	0.032
	Detection of undigested dosage forms (yes vs. no)^a^	16.77	4.05–96.04	< 0.001
	Change of dosage form (yes vs. no)^a^	2.65	0.10–81.11	0.587
	Age (years)	0.999	0.942–1.060	0.980
	Sex (male vs. female)	0.606	0.099–3.714	0.588
Undigested dosage forms	Ostomy type (ileostomy vs. colostomy)	0.085	0.015–0.478	0.005
	Information provided by healthcare professionals (no vs. yes)	0.046	0.005–0.452	0.009
	Communication with physician regarding medication issues (yes vs. no)	9.610	1.262–73.301	0.029
	Age (years)	1.051	0.982–1.125	0.147
	Sex (male vs. female)	0.258	0.032–2.061	0.204

a Estimates derived from Firth penalized logistic regression due to small sample size and sparse data.

Occurrence of undigested dosage forms: Three significant predictors were identified. Ostomy type showed a strong association (*p* = .005, OR = 0.085, 95% CI: 0.015–0.478), corresponding to higher odds for ileostomy patients. Information provided by healthcare professionals was a highly relevant predictor (*p* = .009, OR = 0.046, 95% CI: 0.005–0.452); patients without professional guidance showed increased odds of observing undigested medication residues. Communication with a physician regarding potential medication-related issues was also significant (*p* = .029, OR = 9.610, 95% CI: 1.262–73.301). Age and sex were not significantly associated with the occurrence of undigested dosage forms (age: *p* = .142; sex: *p* = .229).

Association between dosage form variables and perceived effectiveness: To further examine the relationship between dosage form-related variables and perceived medication effectiveness, a binary logistic regression model using Firth's penalized likelihood method was calculated. Detection of undigested dosage forms emerged as a significant predictor (B = 2.819, *p* < .001, OR = 16.77, 95% CI: 4.05–96.04), indicating that participants who observed undigested medications had higher odds of reporting altered medication effectiveness. Change of dosage form was not a significant predictor when controlling for detection of undigested dosage forms (B = 0.973, *p* = .587, OR = 2.65, 95% CI: 0.10–81.11).

## 4 Discussion

This cross-sectional exploratory survey highlights key medication-related challenges in the pharmacotherapy of individuals living with an ileostomy or colostomy. Although the majority of respondents did not report noticeable changes in medication effectiveness, a relevant proportion described experiences suggesting potential medication-related issues, including altered drug effects, the observation of undissolved dosage forms, and insufficient professional counseling.

### 4.1 Information gaps and communication deficits

Among respondents, only 24.5% reported being informed by healthcare professionals about potential changes in medication absorption or effectiveness following ostomy formation, consistent with prior reports on insufficient patient education at care transitions.^26^, ^27^ In the logistic regression analyses, lack of professional information emerged as a significant predictor of both perceived medication effectiveness changes and undigested medication residues. However, the extremely wide confidence intervals—spanning more than two orders of magnitude—reflect the small sample size and indicate substantial uncertainty in these estimates. These findings should therefore be interpreted as hypothesis-generating rather than confirmatory.

The cross-sectional design precludes causal inference. While the data suggest that insufficient patient education may be associated with patient-reported medication problems, reverse causality is equally plausible: patients who experienced medication problems may have subsequently sought information, or both phenomena may share common underlying factors such as disease severity, healthcare engagement, or access to specialized care. Prospective studies with larger samples are needed to clarify these relationships.

Against this background, and in line with current evidence, professional counseling for ostomy patients should specifically address the following aspects:

1. Mechanism: Explanation that ostomy formation, particularly ileostomy, reduces absorptive surface area and transit time, potentially affecting medication absorption^12^, 28.
2. High-risk dosage forms: Identification of sustained-release, delayed-release, enteric-coated, and soft capsule formulations as potentially problematic, with consideration of alternative dosage forms such as liquids, immediate-release formulations, or transdermal systems when appropriate14., 29
3. Monitoring: Instruction to periodically check ostomy bags for undigested medication residues^16^
4. Communication: Emphasis on informing all healthcare providers about ostomy status during consultations^30^
5. Vigilance: Encouragement to report perceived changes in medication effectiveness, even in the absence of visible undigested medications

### 4.2 Medication-related challenges after ostomy formation

Of 48 respondents, 35 (72.9%) reported regular medication use, and 14 (29.2%) reported perceived changes in effectiveness. Additionally, 19/53 participants (35.8%) reported observing undigested tablets or capsules in their ostomy bag. This was significantly more prevalent among ileostomy patients compared with colostomy patients (55.2% vs. 14.3%; *p* = .003). Regression analyses supported ostomy type as a key determinant of both undigested dosage forms and perceived changes in medication effectiveness, again, with wide confidence intervals. These findings are consistent with the understanding that among post- ostomy anatomical changes, the remaining absorptive surface area is the most decisive factor for drug absorption.^31^ Patients with more proximally placed ostomies and shorter distal bowel segments are therefore at increased risk of absorption disorders, which explains why ileostomy patients experience deficiencies in the absorption of nutrients, fluids, and medications significantly more often than colostomy patients.^12^, 13., 14.

The prevalence of undigested medications in this study (35.8%) is slightly higher than previously reported by Remane et al. (22.0%), likely reflecting the larger proportion of ileostomy patients in our sample.^16^ These observations indicate incomplete drug release or absorption, which may result in subtherapeutic exposure—a clinically relevant concern especially for medications with narrow therapeutic windows or essential chronic therapies.

### 4.3 Role of healthcare professionals

While 79.6% of respondents informed their physicians about their ostomy during medical consultations, only 42.9% disclosed this information to pharmacy staff. Discussions specifically about potential medication-related changes with physicians occurred infrequently, suggesting that ostomy status is mentioned but medication-specific risks are rarely addressed.

Physicians were the most frequently cited contact persons for medication-related questions, followed by pharmacists. The involvement of physicians in medication safety management is essential, as they hold primary responsibility for medication therapy.^32^ Given their expertise in dosage forms, drug absorption, and medication safety, pharmacists may play a particularly important role in identifying and preventing ostomy-related medication problems. However, the results suggest that this potential is not yet systematically utilized. This represents a missed opportunity, as clinical pharmacists could help address these issues through proactive screening and individualized counseling.^33^, ^34^

The regression analyses revealed an apparently paradoxical finding: patients who actively discussed medication-related concerns with their physician had higher odds of reporting undigested dosage forms, although estimates were imprecise. This association likely reflects a reactive rather than causal relationship. Patients who observed undigested medications may have been prompted to initiate discussions with their physicians. Additionally, patients actively engaged in such discussions may be taking more complex or critical medication regimens, further increasing the likelihood of observing absorption issues, or may be more attentive to their ostomy output. This pattern suggests that concerns are typically raised after problems have already occurred, rather than receiving anticipatory guidance to prevent or minimize such problems.

### 4.4 Alternative dosage forms as a safety strategy

Of the 49 participants who responded, 14 (28.6%) reported the use or recommendation of alternative dosage forms, such as liquids, orodispersible tablets, or transdermal systems. These alternatives were often perceived as more effective or better tolerated. This finding supports existing pharmacological considerations that non-oral or immediately bioavailable oral formulations may reduce absorption-related risks in patients with altered gastrointestinal anatomy.^35^, ^36^ In contrast, high-risk dosage forms may not fully disintegrate or release their active substance before excretion in patients with an ostomy, particularly those with an ileostomy, due to shortened intestinal length and accelerated transit.14., 29

While bivariate analysis showed a strong association between dosage form changes and perceived effectiveness changes (85.7% vs. 20.6%, *p* < .001), this association was no longer significant when controlling for detection of undigested dosage forms in the regression model. This suggests that dosage form changes were primarily reactive—occurring in response to observed absorption problems—rather than preventive. In other words, patients who detected undigested medications subsequently switched to alternative dosage forms, explaining the bivariate association. This interpretation is consistent with the qualitative findings, where several participants reported switching from tablets to liquid formulations after observing undigested medications in their ostomy bag.

While our data suggest dosage form switching was primarily reactive, existing clinical evidence supports proactive counseling on high-risk formulations to prevent absorption problems before they occur.^16^, ^35^

However, the relatively low uptake suggests that alternative dosage forms are not consistently considered or discussed in routine care, representing another opportunity to improve medication safety.

### 4.5 Strengths and limitations

This study focuses on patient-reported experiences in individuals living with an ostomy, a population that remains underrepresented in patient safety research. By capturing real-world experiences, the study identifies safety-relevant issues—such as undigested dosage forms and perceived changes in medication effectiveness—that are unlikely to be detected through routine clinical data alone.

The standardized and anonymous online survey design ensured consistent data collection and may have encouraged honest reporting. The questionnaire was developed based on relevant literature and refined through pretesting with clinical pharmacists and a patient representative, supporting content validity. The inclusion of both quantitative and qualitative data allowed for a comprehensive understanding of medication-related issues and patient perspectives.

Several limitations must be acknowledged. The cross-sectional design and reliance on self-reported data may have introduced recall and reporting bias. Patients' perceptions of medication effectiveness may not accurately reflect actual pharmacokinetic changes, and the absence of objective measures (e.g., drug levels, clinical outcomes) precludes confirmation of patient-reported effects. The absence of data on ostomy duration, indication for surgery, and comorbidities limits the ability to contextualize experiences. Recruitment through self-help groups may limit generalizability. The voluntary nature of participation may have introduced self-selection bias, potentially leading to an overrepresentation of individuals who experienced medication-related problems. The sample showed a gender imbalance (75.5% female vs. 24.5% male), which differs from the typical male predominance in colorectal cancer, the most common indication for ostomy surgery.^1^, ^37^ While logistic regression analyses showed no significant gender differences in outcomes, the small number of male participants limits the power to detect such differences, and this imbalance may affect generalizability.

The small sample and low event counts resulted in logistic regression models with fewer than 10 events per predictor variable, below recommended thresholds for stable estimation. This is reflected in the wide confidence intervals observed for several odds ratios, indicating substantial uncertainty in effect estimates. Given these limitations, the results should be considered exploratory and used to inform future research rather than as conclusive evidence.

## 5 Conclusion

Patients living with an ileostomy or colostomy report experiences that indicate relevant medication- related challenges warranting clinical attention.

These findings highlight the need for systematic medication review following ostomy formation, proactive patient education on high-risk dosage forms, and consistent documentation of ostomy status across healthcare settings. Pharmacists, in collaboration with physicians, nurses, and ostomy care specialists, are well positioned to contribute to safer pharmacotherapy through targeted counseling and individualized dosage form selection.

Given the exploratory nature of this study and the limited sample size, future research with larger cohorts and objective pharmacokinetic measurements is warranted to confirm these findings and to develop evidence-based guidelines for medication management in ostomy patients.

## CRediT authorship contribution statement

**Stefanie Hehenberger:** Writing – original draft, Visualization, Methodology, Formal analysis, Data curation, Conceptualization. **Sonja Glamočlija:** Investigation. **Irene Lagoja:** Writing – review & editing, Validation, Supervision, Resources, Project administration, Methodology, Conceptualization.

## Ethical considerations

The Ethical Committee of the University of Vienna confirmed that ethical approval was not required for this study (approval confirmation dated 28 May 2025).

## Funding

This research did not receive any specific grant from funding agencies in the public, commercial, or not-for-profit sectors. Open access publication fees were covered by the University of Vienna.

## Declaration of competing interest

The authors declare that they have no known competing financial interests or personal relationships that could have appeared to influence the work reported in this paper.

## Data Availability

Data are available upon reasonable request to the corresponding author.

## Appendix A Supplementary data

Supplementary material Supplementary File 1- Questionnaire
