# Supplementary material for: Medication-related challenges in patients living with an ostomy: Insights from a national cross-sectional exploratory survey
Source: Explor Res Clin Soc Pharm. 2026 May 12;23:100797. doi: 10.1016/j.rcsop.2026.100797 (PMC13197789; doi:10.1016/j.rcsop.2026.100797)
Supplement: Supplementary material — Supplementary File 1- Questionnaire [file mmc1.docx]

Supplementary File 1. Study questionnaire (original German version used for data collection; English version provided for transparency and international readability and not used for data collection).

**Medikation und Arzneimitteleinnahme bei Stoma Patient*innen**

1. **Welche Art von Stoma haben Sie?**□ Ileostoma (Dünndarmausgang)
   □ Kolostoma (Dickdarmausgang)
   □ weiß ich nicht
2. **Geschlecht:**□ weiblich □ männlich □ divers
3. **Alter**: ________ Jahre

**4. Nehmen Sie aktuell regelmäßig Medikamente ein?**

□ Ja □ Nein

Falls ja, wie viele verschiedene Medikamente nehmen Sie täglich ein? ___________________________________________________________________________

**5. Hat sich Ihre Medikation nach der Stomaanlage verändert?**

□ Ja □ Nein

Falls ja, welche Medikamente wurden geändert oder abgesetzt? ___________________________________________________________________________

**6. Haben Sie das Gefühl, dass sich die Wirkung Ihrer Medikamente nach der Stomaanlage verändert hat?**

□ Ja □ Nein

Falls ja, welche Veränderungen haben Sie bemerkt? (z. B. schwächere/schnellere Wirkung, Nebenwirkungen)

___________________________________________________________________________

**7. Wurden Sie von medizinischem Fachpersonal über mögliche Veränderungen der Medikamentenwirkung aufgrund der Stomaanlage informiert?**

□ Ja □ Nein

Falls ja, wer hat Sie darauf hingewiesen?

□ Arzt/Ärztin □ Apotheker*in □ Pflegekraft □ sonstige

**8. Kontrollieren Sie regelmäßig Ihren Stomabeutel auf Rückstände von Medikamenten?**

□ Ja □ Nein

**9. Haben Sie schon einmal (oder mehrmals) offensichtlich unverdaut ausgeschiedene Tabletten, Kapseln oder andere Arzneiformen im Stomabeutel gefunden?**

□ Ja □ Nein

Falls ja, konnten Sie nachvollziehen, um welches Medikament es sich gehandelt hat?

□ Ja □ Nein

Wurde daraufhin Ihre Medikation angepasst oder umgestellt?

□ Ja □ Nein

Falls ja: Welche Anpassungen wurden vorgenommen? ___________________________________________________________________________

**10. Haben Sie mit Ihrem Hausarzt/Hausärztin oder Facharzt/Fachärztin über mögliche**

**Veränderungen der Medikamentenwirkung nach der Stomaanlage gesprochen?**

□ Ja □ Nein

**11. Weisen Sie bei einem Arztbesuch darauf hin, dass Sie ein Stoma haben?**

□ Ja □ Nein

**12. Weisen Sie beim Kauf von Medikamenten in der Apotheke darauf hin, dass Sie ein Stoma haben?**

□ Ja □ Nein

**13. An wen wenden Sie sich, wenn Sie Fragen hinsichtlich Ihrer Medikation haben?**

□ Arzt/Ärztin □ Apotheker*in □ Pflegekraft □ sonstige

**14. Haben Sie andere Darreichungsformen als Tabletten (z. B. flüssige Medikamente, Schmelztabletten) ausprobiert oder empfohlen bekommen?**

□ Ja □ Nein

13. Falls ja: Welche? ___________________________________________________________________________

**15. Gibt es bestimmte Medikamente oder Wirkstoffe, die Sie seit der Stomaanlage nicht mehr vertragen oder vermeiden?**

□ Ja □ Nein

15. Falls ja: Welche? ___________________________________________________________________________

**16. Welche weiteren Erfahrungen haben Sie mit Ihrer Medikation seit der Stomaanlage gemacht? Was sind die größten Herausforderungen?**

___________________________________________________________________________

VIELEN DANK FÜR DIE TEILNAHME AN DIESER BEFRAGUNG!

**Medication and drug use in ostomy patients**

**1. What type of stoma do you have?**

□ Ileostomy (small intestine outlet)

□ Colostomy (large intestine outlet)

□ I don't know

**2. Gender:**

□ Female □ Male □ Diverse

1. **Age:** ________ years

**4. Are you currently taking medication on a regular basis?**

□ Yes □ No

If yes, how many different medications do you take daily? ___________________________________________________________________________

**5. Has your medication changed since your stoma was created?**

□ Yes □ No

If yes, which medications were changed or discontinued? ___________________________________________________________________________

**6. Do you feel that the effect of your medication has changed since your stoma was created?**

□ Yes □ No

If yes, what changes have you noticed? (e.g., weaker/faster effect, side effects)

___________________________________________________________________________

**7. Have you been informed by medical professionals about possible changes in the effect of your medication due to the stoma?**

□ Yes □ No

If yes, who informed you?

□ Doctor □ Pharmacist □ Nurse □ Other

**8. Do you regularly check your ostomy bag for medication residues?**

□ Yes □ No

**9. Have you ever (or on multiple occasions) found tablets, capsules, or other forms of medication in your stoma bag that were clearly undigested?**

□ Yes □ No

If so, were you able to identify which medication it was?

□ Yes □ No

Was your medication adjusted or changed as a result?

□ Yes □ No

If yes: What adjustments were made? ___________________________________________________________________________

**10. Have you discussed possible**

**changes in the effect of your medication after the stoma was created with your family doctor or specialist?**

□ Yes □ No

**11. When you visit the doctor, do you mention that you have a stoma?**

□ Yes □ No

**12. When buying medication at the pharmacy, do you mention that you have a stoma?**

□ Yes □ No

**13. Who do you contact if you have questions about your medication?**

□ Doctor □ Pharmacist □ Nurse □ Other

**14. Have you tried or been recommended other forms of medication besides tablets (e.g., liquid medication, lozenges)?**

□ Yes □ No

13. If yes: Which ones? ___________________________________________________________________________

**15. Are there certain medications or active ingredients that you can no longer tolerate or avoid since having your stoma fitted?**

□ Yes □ No

15. If yes: Which ones? ___________________________________________________________________________

**16. What other experiences have you had with your medication since having your stoma fitted? What are the biggest challenges?**

___________________________________________________________________________

THANK YOU VERY MUCH FOR PARTICIPATING IN THIS SURVEY!
